# Are there lingering effects of Daylight-Saving Time on sleep and health estimates of an Australian population?

**DOI:** 10.1093/sleepadvances/zpag009

**Published:** 2026-03-06

**Authors:** Reece J Kemp, Robert J Adams, Jenny Haycock, Leon C Lack

**Affiliations:** Flinders Health and Medical Research Institute, Sleep Health, College of Medicine and Public Health, Flinders University, Bedford Park, SA 5042, Australia; Flinders Health and Medical Research Institute, Sleep Health, College of Medicine and Public Health, Flinders University, Bedford Park, SA 5042, Australia; Flinders Health and Medical Research Institute, Sleep Health, College of Medicine and Public Health, Flinders University, Bedford Park, SA 5042, Australia; Flinders Health and Medical Research Institute, Sleep Health, College of Medicine and Public Health, Flinders University, Bedford Park, SA 5042, Australia; College of Education, Psychology and Social Work, Flinders University, Bedford Park, SA 5042, Australia

**Keywords:** Daylight-Saving Time, permanent Standard Time, circadian rhythms, Australian population, insomnia, sleep assessment, nationwide survey, circadian misalignment, sleep quality, daytime functioning

## Abstract

Research has examined acute sleep effects at the immediate transition onto Daylight-Saving Time (DST), culminating in a sentiment that it should be abolished. These effects could be theorized to continue well-into the DST period. The current article aimed to address this gap, investigating the effect of DST on sleep during the middle-late stages (2–4 and 6 months) of the DST period. A retrospective, cross-sectional design was used to compare subjective data of two nationwide surveys of the Australian population; one population-representative sample, and a convenience sample of those with chronic Insomnia. Respondents were categorized based on whether they were from a DST state or permanent Standard Time (ST) state. We then compared sleep behavior and tendencies in sleep health between DST and ST states. Overall, both samples consistently demonstrated that those from DST states tended to go to bed later and, particularly, rise at later clock times than those from ST states. Importantly, despite a delay in the timing of sleep we found no differences in reported Total Sleep Time nor Sleep Onset Latency; and no sign of impairment on any related health estimates. Very few sleep health variables reached significance (*p* < .05), and the vast majority of them suggested those from DST states were *less* impaired than their ST counterparts. We have found no evidence of impairment associated with DST well-into the DST period. Future studies should measure sleep and associated daytime functioning longitudinally and objectively to accurately assess the possible duration of any potential acute DST effect.

Statement of Significance The research presented here offers a valuable expansion to the sleep and health effects of Daylight-Saving Time (DST). To our knowledge, it is the only population-level study examining DST effects well-into the DST period, as well as one of the few studies to comprehensively assess sleep and daytime functioning measures between DST and Standard Time conditions. The study has found no lingering detrimental effects of DST on subjective measures of sleep or associated daytime functioning. This raises the need for an objective and longitudinal study of sleep across the DST transition period to accurately assess the health cost of the transition onto DST.

## Introduction

Across the globe, over 1.3 billion people spanning numerous countries observe a seasonal practice known as “Daylight-Saving Time” (DST), which gives an additional 1 h of natural light in the evening during the summer period [1]. Recently, there has been a steady growth of literature linking the transition onto DST to various health risks, including acute cardiovascular issues, mood disorders, motor vehicle accidents, and impaired psychomotor vigilance [1–4]. These risks are often attributed to a disruption of sleep patterns induced by the clock change of DST. Numerous studies have identified that the transition onto DST results in a significant reduction in sleep duration, a phenomenon observed across different regions, age groups, clinical populations, and even simulated estimates of sleep behavior based on energy usage [5–9]. The general consensus is that these effects are of a considerable detriment to those experiencing DST.

In response to this, recent position statements from research societies have called for the elimination of DST due to its potential adverse effects on health. Bodies such as the Sleep Research Society have published a position statement supporting the removal of DST [10, 11]. The American Academy of Sleep Medicine and other bodies have also begun advocating for its abolition, citing the detrimental impact of DST on individuals’ health [12, 13].

**Table 1:** Demographic variables for study 1 (population sample) including age, body mass index, rurality, and sex between DST and ST states

Demographic Variable		State		Inferential statistic
		ST (*n* = 303)	DST (*n* = 704)	
Age^*^		47.51 (17.12)	44.95 (15.92)	*t*(536.51) = 2.22, *p* = .03*d* = 0.16 [0.02–0.29]
Body mass index (BMI)^*^		28.17 (6.32)	27.51 (7.03)	*t*(869) = 1.28, *p* = .20*d* = 0.10 [−0.05–0.24]
Income^*^		$75 901 ($48 196)	$78 882 ($49 938)	*t*(853) = −0.81, *p* = .42*d* = −0.60 [−0.21–0.09]
Rurality^†^	Metropolitan	198 (65.30%)	483 (68.60%)	*χ^2^*(1, 1007) = 0.89
	Rural	105 (34.70%)	221 (31.40%)	*p* = .35, *φ* = −.032^‡^
Sex^†^	Male	141 (46.50%)	360 (51.10%)	*χ^2^*(1, 1007) = 1.62
	Female	162 (53.50%)	344 (48.90%)	*p* = .20, *φ* = −.041^‡^
Education^†^	Still at school	4 (1.30%)	18 (2.60%)	χ^2^(7, 995) = 25.90
	Left school at 16 or less	41 (13.70%)	53 (7.60%)	*p* < .001, *φ* = .16
	Left school after 16	42 (14.00%)	81 (11.60%)	
	Left after 16 but still studying	6 (2.00%)	15 (2.20%)	
	Trade/apprenticeship	22 (7.40%)	38 (5.50%)	
	Certificate/diploma	109 (36.50%)	217 (31.20%)	
	Bachelor’s degree or higher	75 (25.10%)	274 (39.40%)	

^*^Independent sample *t*-test reports mean (standard deviation).

^†^Chi-square test reports frequency (relative percentage to *N*).

^‡^After Yates’ continuity correction.

However, the documented sleep impacts have only been reported for the immediate or early transition onto DST. Whether these effects alleviate or are still present over time is not known. The continued (or chronic) impairment associated with DST across the summer period has largely been overlooked in the literature. Prior work and importantly the European Union have identified this gap in the literature and called for the “full evaluation” of the effect of DST [12–14]. Additionally, the literature has predominantly focused on reduction in sleep duration as the main impact and cause of other impairments. A more comprehensive analysis of the circadian/sleep and daytime functioning impacts of DST is necessary to evaluate the total impairment produced by DST.

Therefore, the current study addresses the question of any impairment attributable to DST past the initial transition, focusing on the middle-later parts of the DST period. We make use of Australia as the basis for our analyses, given that some states in Australia stay on Standard Time (ST) throughout the year, while some states observe DST for 6 months including the Summer period. Australia, despite the opportunity of co-existing ST and DST states, has had relatively few published studies on DST, and even fewer which assess its impact on sleep [15–17]. To our knowledge, there has been only one other study that has assessed DST in relation to sleep [18]. This study, which longitudinally examined differences in average sleep duration across the year within a DST state, found *no* significant difference in Total Sleep Time (TST) at the transition onto DST, which is inconsistent with most of the global literature. This raises the need to examine the effects of DST on sleep more comprehensively, particularly in an Australian population. We report on two studies of the Australian Population, the first being a population-representative sample, and secondly a convenience sample of Australians presenting with chronic insomnia symptoms [19, 20]. These two samples will allow us to make inferences at a population level, but also evaluate the total impact of DST in an insomnia population which may be more sensitive to the possible disruptive effects of DST [8].

## Study 1—Population sample

### Method

This sample comprised of a population-representative survey of 1007 participants (506 females; 501 males) responding in March 2016, the final month of the DST period [19]. Four participants who reported their postcode as “9999; *Don’t Know*” were excluded from the analysis. Eligible participants were then coded based on whether they resided in states of Australia that use DST (New South Wales, Australian Capital Territory, Victoria, South Australia, and Tasmania) during the summer months (*n* = 704) or in states (Queensland, Northern Territory, and Western Australia) that stay on ST throughout the year (*n* = 303).

A retrospective cross-sectional design was used to examine DST’s impact on reported sleep timing variables, including bedtimes and waketimes on workdays and freedays; TST, Sleep Onset Latency (SOL), and extension of sleep on freedays (TST_freeday_ − TST_workday_). We distinguished between sleep on workdays and freedays (in addition to an average nightly measure) in accordance with previous literature [5, 7, 8].

The survey included many estimates of sleep health and daytime functioning measures, including the Epworth Sleepiness Scale (ESS), Stanford Presenteeism Scale (SPS-6), drowsy driving, and a range of subjective estimates of sleep quality and daytime performance.

**Figure 1:**
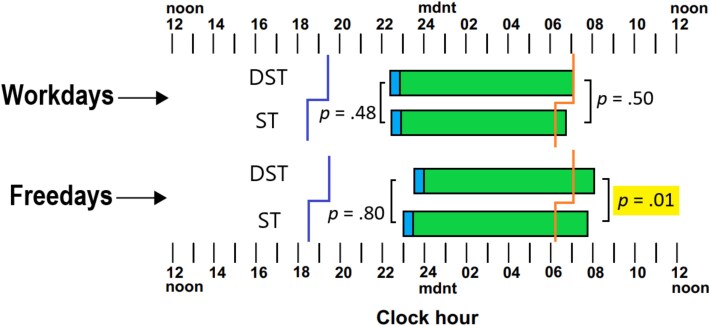
Sleep timing of the population sample; including sunset (purple)/sunrise (orange) times, bedtimes (left), Sleep Onset Latency (blue), Total Sleep Time (green), and Waketimes (right), between workdays and freedays, Daylight-Saving Time (DST), and Standard Time (ST) states. Original figure created by the author.

**Table 2:** Estimated marginal (EM) mean (*SE*) sleep variables available in study 1 including bedtime and waketime (in 24-h decimals), total sleep time (hours/h) for workdays, freedays (including sleep extension [minutes/min]), and nightly (including sleep onset latency [minutes/min]) between standard time (ST) and daylight-saving time (DST) states

Daytype	Sleep behavior	State		Inferential Statistic
		ST	DST	
Workday	Bedtime	22.48 (0.30)	22.30 (0.19)	*F*(1, 925) = 0.49*p* = .48, η_p_^2^ = .001
	Waketime	6.87 (0.26)	7.03 (0.16)	*F*(1, 946) = 0.45*p* = .50, η_p_^2^ = .000
	Total Sleep Time (h)	7.00 (0.19)	7.03 (0.10)	*F*(1, 898) = 0.02*p* = .92, η_p_^2^ = .000
Freeday	Bedtime	23.00 (0.31)	23.10 (0.19)	*F*(1, 967) = 0.06*p* = .80, η_p_^2^ = .000
	Waketime	7.44 (0.24)	8.19 (0.15)	*F*(1, 946) = 6.78*p* = .01, η_p_^2^ = .01
	Total Sleep Time (h)	7.49 (0.18)	7.63 (0.11)	*F*(1, 893) = 0.33*p* = .57, η_p_^2^ = .000
	Sleep extension (min)^a^	36.60 (1.5)	36.78 (0.08)	*F*(1, 882) = .003*p* = .96, η_p_^2^ = .000
Nightly	Sleep Onset Latency^b^	N/A	0.86 (0.50)	*χ^2^*(1) = 2.94, *p* = .09OR = 2.37 [0.88–6.36]
	Total Sleep Time (h)	7.19 (0.19)	7.20 (0.10)	*F*(1, 882) = .003*p* = .96, η_p_^2^ = .000

^a^Levene’s test indicated unequal variances (*p* = .003).

^b^Ordinal regression was used to determine SOL due to ordinal categorical raw data. Descriptive statistic = *β* (*SE*), ST state comparisons become redundant.

There were small but significant differences amongst some demographic variables (see Study 1: Results); hence, in order to evaluate differences in the following outcome variables between DST and ST states while controlling for age and education level we implemented Analysis of Covariance (ANCOVA) for all sleep behavior variables. We also note here that despite differences in rurality being non-significant, we controlled its potential effects in conjunction with the other measures listed above, and have included it in the analysis as a covariate.

The ESS was used to measure subjective daytime sleepiness. Responders rated the likelihood of dozing off in various scenarios, and scores were combined to determine daytime sleepiness severity, ranging from normal (0–5) to severe (16–24) [21]. Additionally, the SPS-6 was employed to assess workplace presenteeism, measuring perceived productivity and work quality at work despite potential sleep disturbance. Higher SPS-6 scores in this context indicated better daytime functioning despite poor sleep [22].

Ordinal categorical variables were primarily used to investigate subjective estimates of sleep health, arranged such that higher scores indicated higher frequency (e.g. 1 = “*rarely or never*” to 4 = “*every night or almost every night*”); unless, it was a binary categorical scale (e.g. 1 = “*yes,”* 2 = “*no”*). Ordinal and Binary logistic regressions were conducted to examine the effect of state on all categorical sleep outcomes, while controlling for age, education, and rurality. Results were expressed as parameter estimate *β* (standard error [*SE*]), and the Wald (*χ*^2^) statistic was used to infer the significance of each predictor as well as odds ratios (OR, 95% confidence intervals [CIs]).

### Results

In terms of demographic information, we were able to assess age, body mass index (BMI), income, rurality, sex, and education. Significant differences in age have been reported in Table 1. Those from DST states were significantly younger than those from ST states. We also report that those from DST states attained a higher educational level on-average compared to those from ST states.

Figure 1 illustrates mean sleep timing and duration on workdays and freedays (e.g. weekends) between those responding from DST states and those from ST states. It illustrates the data described in Table 2 on a 24-h clock with the sunset/sunrise times between DST states and ST states.

As Figure 1 and Table 2 show, those responding from DST states report, on-average, a trend toward earlier bedtimes on workdays, later bedtimes on freedays, and later waketimes for both workdays and freedays, although the only effect to reach significance was waketimes on freedays. Those from DST states were rising significantly later than those from ST states (by ~45 min).

It can be seen that there was no difference in extension of sleep on freedays for DST states compared to ST states. Respondents from either DST or ST states were sleeping longer on freedays by about 36 min.

Table 2 also shows the mean reported TST between those from DST and ST states. Evidently, those from DST states reported slightly *more* sleep compared to those from ST states, though these differences were not significant and reflected negligible effect sizes. On workdays, regardless of state, individuals were sleeping for around 7 h, and just over seven and-a-half hours on freedays. Averaging workdays and freedays together appropriately weighing them by 5 and 2, respectively, nightly TST across the total week showed no difference between DST and ST states. Finally, there were no differences in SOL between DST and ST states. While there was a trend for those from DST states to have shorter SOL, on average, most people reported taking between 10–15 and 15–30 min to fall asleep.

Turning toward sleep health and daytime functioning measures, Table 3 shows the sleep questionnaire measures we investigated. There was no significant difference of daytime sleepiness (ESS score) between states, and both scores reflected “normal” daytime sleepiness. We analyzed the SPS-6 and as Table 3 shows, there were no significant differences between ST and DST states for the SPS-6, with respondents from both states being able to competently work despite possible sleep disturbance.

**Table 3:** EM mean (*SE*) scores on questionnaire data available in study 1 including epworth sleepiness scale (ESS) and stanford presenteeism scale (SPS-6)

Scale	State		Inferential statistic
	ST	DST	
Epworth Sleepiness Scale	7.03 (0.50)	6.42 (0.31)	*F*(1, 967) = 0.54* p* = .46, η_p_^2^ = .001
Stanford Presenteeism Scale 6^*^	20.12 (0.62)	20.92 (0.51)	*F*(1, 442) = 1.60 *p* = .21, η_p_^2^ = .0024

^*^Levene’s test indicated unequal variances (*p* = .005).

**Table 4:** Logistic and ordinal regression analyses (*β* [*SE*]) predicting the probability of higher impairment in drowsy driving measures, and a variety of sleep health measures available in study 1 for DST states

Measure	DST state probability *β* (*SE*)	Inferential statistic
Driven while asleep^*^	0.40 (0.37)	*χ^2^*(1) = 1.17, *p* = .28 OR = 1.50 [0.72–3.10]
Had accident while drowsy	0.38 (0.54)	*χ^2^*(1) = 0.49, *p* = .48OR = 1.46 [0.50–4.25]
Accident frequency	−2.26 (2.89)	*χ^2^*(1) = 0.61, *p* = .44 OR = 0.11 [0.0004–30.19]
Interfering sleepiness	1.25 (0.51)	*χ^2^*(1) = 6.02, *p* = .01 OR = 3.49 [1.29–9.39]
Daytime fatigue	0.85 (0.50)	*χ^2^*(1) = 2.93, *p* = .09 OR = 2.33 [1.09–6.15]
Irritability^†^	0.79 (0.50)	*χ^2^*(1) = 2.46, *p* = .12 OR = 2.20 [0.82–5.90]
Difficulty falling asleep^†^	0.95 (0.50)	*χ^2^*(1) = 3.68, *p* = .06 OR = 2.59 [0.98–6.82]
Frequent awakenings	1.42 (0.49)	*χ^2^*(1) = 8.38, *p* = .004 OR = 4.15 [1.58–10.87]
Waking unrefreshed^†^	0.89 (0.49)	*χ^2^*(1) = 3.22, *p* = .07 OR = 2.42 [0.92–6.37]
Early morning awakening	0.12 (0.49)	*χ^2^*(1) = 0.06, *p* = .82 OR = 1.12 [0.43–2.96]

^*^Binary categories (yes/no).

^†^Test of parallel lines indicated skewed proportions for: Difficulty falling asleep *p* = .005; waking unrefreshed *p* = .04; accident frequency *p* = .001.

Table 4 reports the predicted tendency to indicate poorer sleep health and daytime functioning utilizing ST states as the reference group. Positive *β* in this instance reflected a decreased likelihood of impairment in DST states, and odds ratios < 1 indicate increased chances of scoring higher on these outcomes for DST states. As Table 4 shows, we obtained no evidence that those from DST states had any sleep related driving impairment relative to ST states, in fact the general trend indicated that ST states fared worse than those from DST states, although non-significant. Most respondents reported driving while asleep “less than once per month” to “never.” Also, there were no differences in reported accidents or accident frequency between states.

As for the rest of the measures, we did obtain significant effects for sleepiness that interferes with daytime activities (Interfering Sleepiness), and Frequent Awakenings, in which those from DST states had a *lower* probability of experiencing greater impairment compared to ST states. Aside from that, there were no significant tendencies between states on any of the self-reported sleep quality measures, albeit with a general tendency for DST states to report slightly less impairment. Though, with most ORs being quite wide, these trends are mostly trivial.

## Study 2—Insomnia sample

### Method

This dataset was a convenience sample of 390 individuals who had responded to publicity about insomnia treatment. We have reported on individuals who had an Insomnia Severity Index (ISI) score of 15 or greater (a common threshold for clinical insomnia), and five participants who provided invalid postcodes (e.g. PO boxes) were excluded from the analysis. Additionally, given that the prevalence for insomnia among shift workers is quite considerable [23], we have excluded any participants that reported themselves as a shift-worker (*n* = 24) as to avoid any potential for shift-work to confound with the sleep timing/health measures we have investigated. This questionnaire was sent to them between November 2021 and January 2022, around the middle of the DST period [20]. This sample was predominantly female (70.17%) with an average age of 55.27 years (*SD* = 13.26). Again, a majority (*n* = 335) were from DST states, and 55 individuals were from ST states.

**Figure 2:**
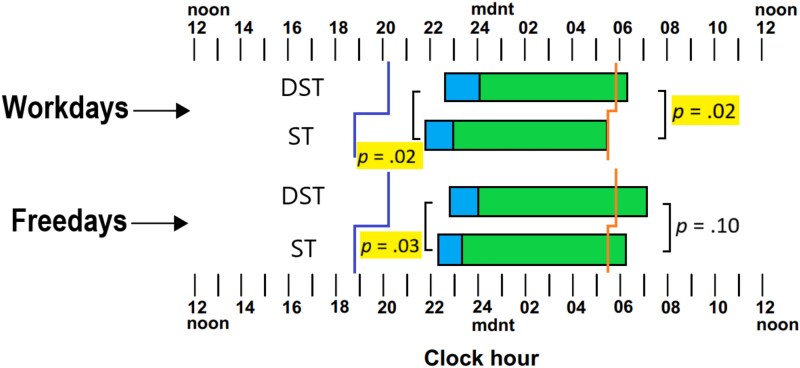
Sleep timing of the insomnia sample; including sunset (purple)/sunrise (orange) times, bedtimes (left), Sleep Onset Latency (blue), Total Sleep Time (green), and Waketimes (right), between workdays and freedays, Daylight-Saving Time (DST), and Standard Time (ST) states. Original figure created by the author.

**Table 5:** EM mean (*SE*) of sleep variables for study 2 (insomnia sample) including bedtime and waketime (in 24-h decimals), total sleep time (hours/h) for workdays, freedays (including sleep extension [minutes/min]), and nightly (including sleep onset latency [minutes/min]) between standard time (ST) and daylight-saving time (DST) states

Daytype	Sleep Behavior	State		Inferential statistic
		ST	DST	
Workday	Bedtime	21.90 (0.23)	22.48 (0.09)	*F*(1, 370) = 5.31*p* = .02, η_p_^2^ = 0.01
	Waketime	5.42 (0.31)	6.20 (0.13)	*F*(1, 347) = 5.29*p* = .02, η_p_^2^ = 0.02
	Sleep Onset Latency (min)	83.64 (11.72)	84.21 (3.63)	*F*(1, 377) = 0.002*p* = .96, η_p_^2^ = .000
	Total Sleep Time (h)	4.77 (0.49)	5.41 (0.20)	*F*(1, 370) = 1.44*p* = .23, η_p_^2^ = .004
Freeday	Bedtime	22.41 (0.30)	23.10 (0.12)	*F*(1, 338) = 4.54*p* = .03, η_p_^2^ = 0.01
	Waketime	6.32 (0.40)	7.04 (0.17)	*F*(1, 313) = 2.78*p* = .10, η_p_^2^ = 0.01
	Sleep Onset Latency (min)	63.98 (10.49)	77.97 (4.17)	*F*(1, 357) = 1.54*p* = .22, η_p_^2^ = .004
	Total Sleep Time (h)	5.41 (0.53)	5.78 (0.21)	*F*(1, 349) = 0.42*p* = .52, η_p_^2^ = .001
	Sleep Extension (min)	31.27 (9.59)	21.04 (3.78)	*F*(1, 347) = 0.99*p* = .32, η_p_^2^ = .003
Nightly	Total Sleep Time (h)	4.98 (0.53)	5.52 (0.21)	*F*(1, 347) = 0.91*p* = .34, η_p_^2^ = .003

A retrospective cross-sectional design was also utilized for this sample. Univariate ANOVAs were utilized for all measures, and there were no significant differences in demographics (age and sex) between states. We examined the same sleep timing measures as in Study 1, as well as available standardized sleep scales (e.g. ESS and ISI). As for the various sleep quality and daytime functioning measures between DST and ST states, we implemented Ordinal Regression Analyses (with same interpretation as Study 1). Once again, these measures were selected based on their potential to be impacted due to DST. Ordinal categorical scales assessed sleep quality and daytime feelings and reflect the same interpretation as Study 1 (higher scores = greater impairment).

The ISI was available to evaluate sleep quality. It corresponds to the diagnostic criteria for insomnia, and item scores are rated from zero to four based on the perceived severity of individual symptoms, with a total score ranging from 0 to 28 [24]. We note here that due to our sample only including individuals with an ISI > 14, this sample’s scores ranged from 15 to 28.

### Results

Similar to Study 1, we have provided a figure that illustrates the mean sleep period for the DST and ST states for workdays and freedays. Figure 2 and Table 5 reveal a similar pattern of results to Study 1 showing a later sleeping pattern in DST states. Bedtimes and waketimes were later in the DST states on workdays and freedays, with only the effect of waketimes on freedays not reaching a significant level.

Table 5 also shows there was no significant difference in reported TST between DST and ST states. While those from DST states had slightly more sleep on workdays and freedays, these differences were non-significant and negligible based on partial eta squared.

Regarding SOL, unlike the population sample (Study 1), those from DST states tended to have longer SOL on both workdays and freedays compared to those from ST states, and a tendency for more sleep per night for the week as a whole. However, none of these differences were significant with negligible effect sizes based on partial eta squared. Furthermore, there was no significant difference in sleep extension between DST and ST states, with responders from DST states sleeping on average about 22 min more on freedays, and 32 min for ST states.

Table 6 reports the scores on standardized questionnaires available in Study 2 (ISI and ESS). We found significant differences in insomnia severity via the ISI, and daytime sleepiness from the ESS. While all effect sizes were negligible small based on partial eta squared, those from DST indicated significantly *lower scores* on the ISI, but significantly *higher* scores on the ESS. Interpretation of these scores infer that regardless of state, individuals typically reported moderate insomnia severity, and normal-moderate daytime sleepiness.

**Table 6:** EM mean (*SE*) scores on questionnaire data available in for study 2 including insomnia severity index (ISI) and epworth sleepiness scale (ESS)

Scale	State		Inferential statistic
	ST	DST	
Insomnia Severity Index	20.18 (0.44)	19.15 (0.18)	*F*(1, 387) = 4.67*p* = .03, η_p_^2^ = 0.01
Epworth Sleepiness Scale	5.51 (0.66)	6.93 (0.27)	*F*(1, 386) = 4.07*p* = .04, η_p_^2^ = 0.01

**Table 7:** Parameter estimate (*β*) and standard error (*SE*) of a variety of sleep health measures available in for study 2 for DST states

Measure	DST state probability *β* (*SE*)	Inferential statistic
Good sleep	0.39 (0.28)	*χ^2^*(1) = 1.95, *p* = .16OR = 1.47 [0.85–2.56]
Enough sleep	0.62 (0.28)	*χ^2^*(1) = 5.09, *p* = .02OR = 1.86 [1.09–3.20]
Sleep worry	0.09 (0.27)	*χ^2^*(1) = 0.10, *p* = .75OR = 1.09 [0.64–1.85]
Sleep control	0.30 (0.27)	*χ^2^*(1) = 1.24, *p* = .27OR = 1.35 [0.80–2.29]
Interfering sleepiness	0.78 (0.27)	*χ^2^*(1) = 8.09, *p* = .01OR = 2.17 [1.27–3.71]
Irritability	0.35 (0.27)	*χ^2^*(1) = 1.78, *p* = .18OR = 1.42 [0.85–2.39]
Hyperactivity	0.58 (0.27)	*χ^2^*(1) = 4.50, *p* = .03OR = 1.78 [1.04–3.06]
Loss of pleasure	0.61 (0.27)	*χ^2^*(1) = 5.19, *p* = .02OR = 1.83 [1.09–3.80]
Depressive symptoms	0.35 (0.26)	*χ^2^*(1) = 1.80, *p* = .18OR = 1.42 [0.85–2.37]
Daytime fatigue	0.34 (0.27)	*χ^2^*(1) = 1.62, *p* = .20OR = 1.41 [0.83–2.38]

The sleep quality measures showed that those responding from both DST or ST states rarely had good sleep, enough sleep; rarely had control over their sleep and often worried about getting a good night’s sleep. The only measure to achieve significance here was in achieving enough sleep per night, which indicated those from ST states were more impaired.

Continuing on, we also achieved significant tendencies in Interfering Sleepiness, Hyperactivity, and Loss of Pleasure; all again indicating that those from ST states were more impaired than their DST counterparts. Finally, there were no significant tendencies between any of the other sleep quality measures between states, albeit with a minor trend of greater impairment in ST states.

## Discussion

We investigated whether there was a detrimental effect of DST on the Australian population during the latter parts of the DST period. Both of our studies consistently demonstrated that the effect of DST was limited entirely to the clock times of sleep, specifically individuals’ bed- and waketimes; those from DST states were going to bed significantly later and rising significantly later than those from ST states. Despite this delay in sleep timing by clock time, we found *no significant reduction* in sleep duration as well as no significant impairments in self-reported sleep quality or daytime performance.

Prior studies have suggested, or at least hinted at a long-term impairment arising from DST [5, 6, 12, 13]. For example, the American Academy of Sleep Medicine (AASM), while acknowledging that there is little evidence on the chronic effects, posit that sleep impairments could continue “even after several months [of the transition]” and, as a permanent period, could result in a “permanent phase delay” and hence “chronic sleep loss” (p. 1782) [12].

Indeed, while no article outright claims long-term impairment, the theoretical mechanisms make it at least plausible. The immediate phase delay placed on individuals upon the transition [25], combined with a propensity to stay out later [9], and mismatched with a truncation of sleep opportunity due to early social commitments technically starting an hour earlier [7, 26, 27], could cumulatively lead to chronic sleep loss particularly during the workweek. This worknight sleep loss may lead to longer and later compensatory sleeping on the weekend, which in turn reduces sleep drive and delays the body clock ahead of the next workweek, thus chronically reinforcing sleep loss possibly throughout the DST period [28, 29].

Our findings suggest otherwise; although those from DST states indeed showed delayed bedtimes, waketimes were also significantly later (ranging from 10 to 45 min), allowing for sufficient sleep opportunity as to avoid any short-changing of TST. Specifically, regarding Australian findings, a study mentioned earlier (see Introduction) found no significant reduction in objectively measured sleep duration following the immediate transition compared to the week prior to the transition [18]. Our results add further support to this finding, demonstrating that at least in Australia, the DST period is not associated with an acute nor prolonged impairment to sleep duration. Perhaps, the DST lighting conditions (sunset/sunrise), primarily the differences in sunrise times which, being close to the strongest phase advance point in the normal phase response curve to light, could influence sleep timing [30, 31]. Thus, those from DST states were able to wake up *late enough* as to avoid any short-changing of sleep and downstream impacts to sleep health or daytime performance. Alluding to this idea can be seen in Figure 1 for the population sample where the waketimes on workdays almost coincide with sunrise times in both DST and ST groups.

Furthermore, we found no difference between DST and ST states in reported SOL or standardized questionnaires of sleepiness (ESS) and daytime performance (SPS-6). Additionally, for Study 1, we analyzed a considerable amount of sleep quality and reported daytime performance (health outcome) measures in the latter parts of the DST period. We observed DST states faring sometimes significantly *better*, but for the most part on-par with those from ST states. The most reasonable explanation is that net difference in nightly TST across the whole week was less than a minute. Taking this into account, it is obviously unlikely that the presence of DST would be accompanied by any reported impact in these sleep affected measures.

One notable observation was that there was no difference in reports of drowsy driving and driving accident history between states. The literature is yet to have consensus on the effect of DST and traffic accidents [32]. Based on our results, we conclude that any impairments observed immediately after the transition were no longer present in the late stages of the DST period. Perhaps, individuals from DST states fully adjusted to a later circadian (sleep/wake) timing in conjunction with extended ambient lighting conditions during the evening peak driving hours. This notion is consistent with previous research into DST which identified that the extended ambient light of the DST period significantly *reduced* instances of motor vehicle accidents [33, 34]. The various changes during DST have been shown to offer some positive/protective effects, and is worth considering in future research to fully evaluate the effect of DST [35].

Study 2 was a convenience sample of a large number of people making enquiries about sleep treatments at our laboratory (Flinders Health and Medical Research Institute [FHMRI]: Sleep Health) who reported above threshold scores on the ISI, a valid measure of clinical insomnia. We explored the idea that if DST had disruptive effects on sleep, this sample of individuals with high ISI scores would show higher impairment. We were admittedly surprised at the general lack of any DST impairment. As in Study 1, the findings for this sample were the several significant effects on sleep timing, which potentially indicates that certain populations (e.g. sleep-disordered sample) may have a more pronounced response to the DST period. However, there was no impairment to TST or sleep health measures in this insomnia sample, except for ESS in which those from DST states scored significantly higher than ST states. Aside from that, like Study 1 the majority of evidence suggested that the insomnia sample from DST states reported (sometimes significantly) *less* daytime impairment than those from ST states including less interfering sleepiness.

There are some notable limitations to this report. Firstly, the conclusions we have presented here are relying on subjective data. Although subjective data is rich in terms of insight into the lived experience under DST, it is without question that it is inherently imprecise data and can be biased. Recall bias, perceptual bias, and variability in respondents’ interpretation of items; all of these factors may blur the exact extent to which DST is impacting our results, and limits the scope to which our findings can be generalized. Secondly, we have used the state of residence as the basis for which we have determined DST/ST exposure. While this approach was pragmatic and efficient, it introduced the problem of heterogeneity between states in terms of light exposure, environmental conditions, social schedules, and so on. Moreover, it is important to note that DST states in Australia are predominantly located in the south and experience substantially larger seasonal changes in photoperiod across the year (e.g. Sydney ≈ 4.5 h; Hobart ≈ 6.5 h) compared with northern ST regions (e.g. Darwin ≈ 1.5 h) [36]; it could be the case that that long-term DST effects in our sample may be confounded by natural differences in daylight exposure across latitude. In all, the use of this sampling method does not fully control for the regional or personal variations within and between DST and ST states.

Nevertheless, the most notable strength of this study is its comprehensiveness. To date, the present analysis has provided one of the most thorough evaluations into the effects of the DST period on sleep and associated health effects in both a population sample and sleep-disordered population. Additionally, our comparison of those from DST and ST conditions *at the same time*, thus controlling for potential seasonal variation lends strength to the results we have reported. We have attempted to integrate several dimensions of sleep behavior and sleep health, that of which are often considered in isolation in the wider literature. Taken together, we are confident that we have provided a reasonable basis for the findings drawn about the relationship between DST and sleep.

In conclusion, our investigation assessed the impact of DST on the Australian population during the middle to latter parts of the DST period. Our results consistently revealed that the effect of DST was limited to the later clock timing of sleep which may be indicative of partial but not full adjustment to DST lighting conditions. Furthermore, our analyses of various sleep health measures revealed no evidence of impairment associated with DST, at least well into the DST period. This strongly suggests that future studies should measure the effects longitudinally and objectively across the entire DST period. Such a study would aim to measure, for the population in general, the duration of the impairments following the transition onto DST and provide a better assessment of the totality of the possible negative effects of DST.

Furthermore, longitudinal studies should also consider individual differences in the transition to DST. For example, research into sleep adjustments from small (one-two hour) changes in schedule showed that, despite the majority of the sample (*N* = 19) adjusting their sleep/wake period within 1–2 days from the change onto DST, a significant minority (36%) failed to adjust over the two weeks post transition onto DST suffering continued reduced TST and increased daytime tiredness [37, 38]. Furthermore, patients diagnosed with delayed sleep/wake phase disorder were found to have reduced TST for those diagnosed during DST compared to those diagnosed during ST [8]. This suggests large individual differences in rate of adjustment with potentially some individuals not fully adjusting and being at increased health risk for a considerable time into the DST summer period. Longitudinal studies that could identify such slow adjustors could also investigate predictors of these phenotypes to allow preventative measures before the DST transition.

## Data Availability

The datasets generated and/or analyzed in the current study are not publicly available due to ethical, privacy, and confidentiality restrictions, but can be available from the corresponding author on reasonable request.
